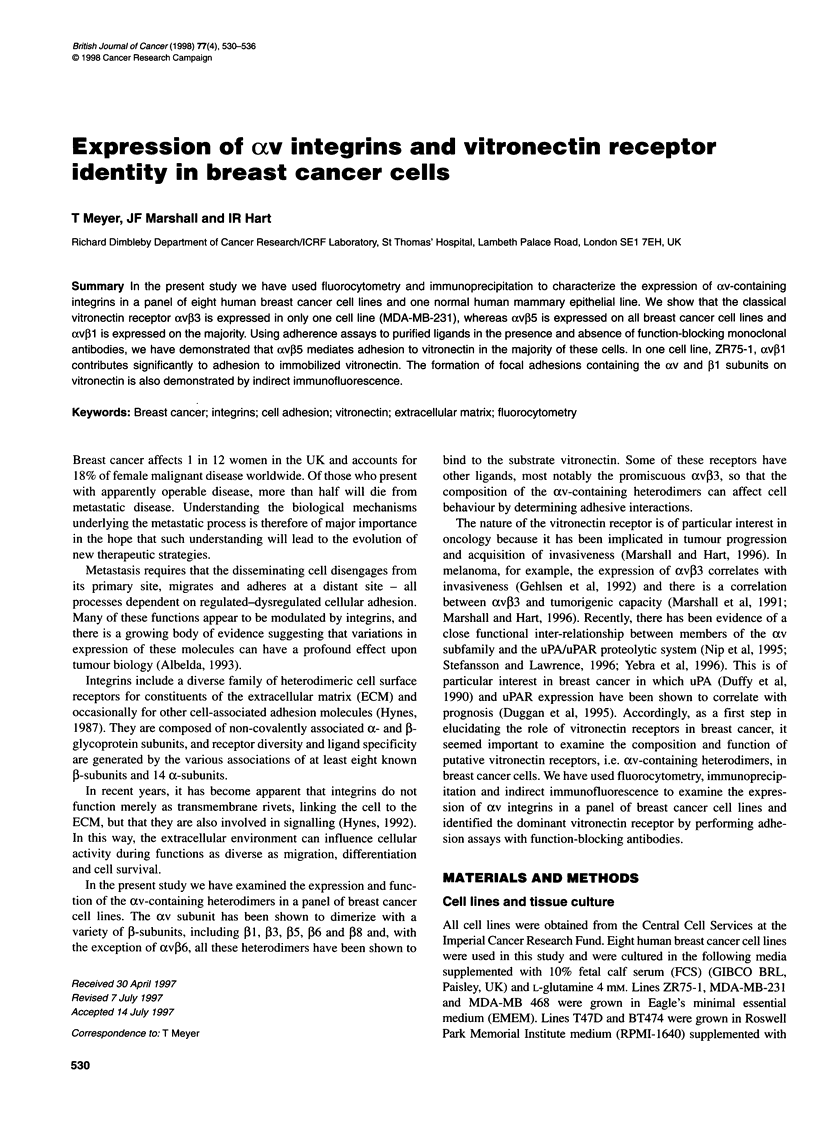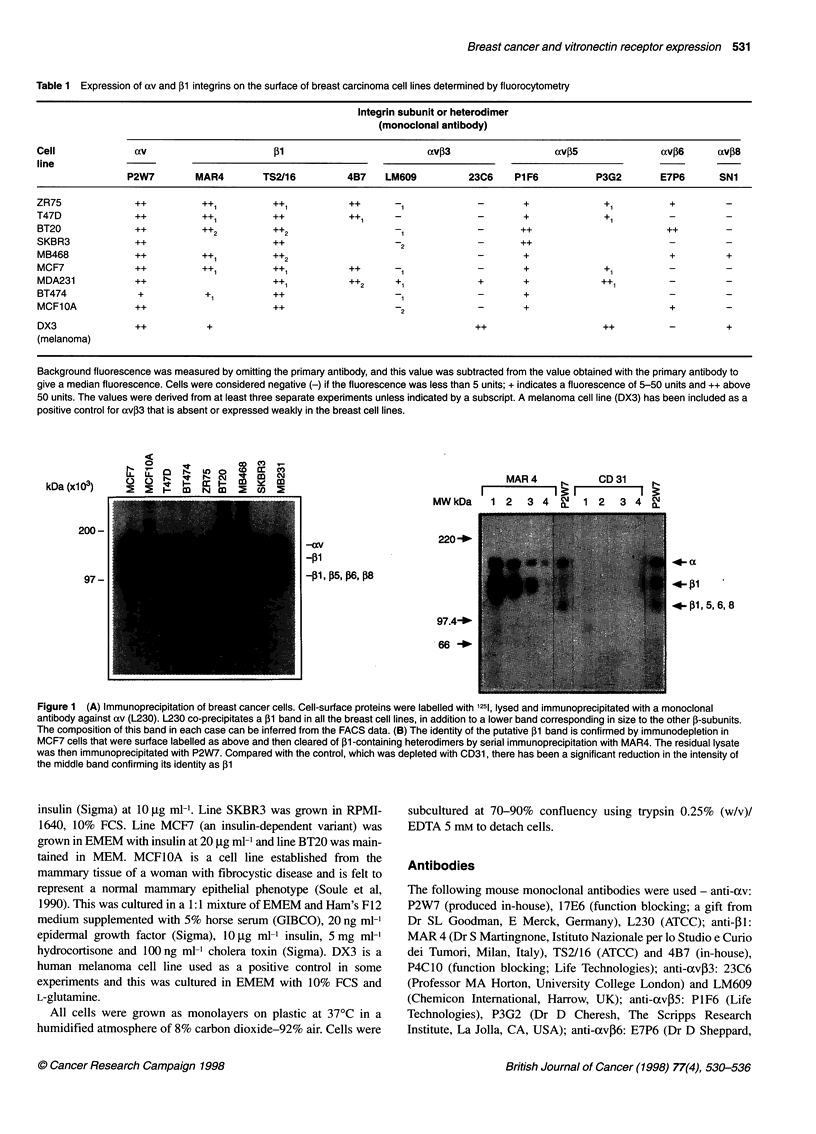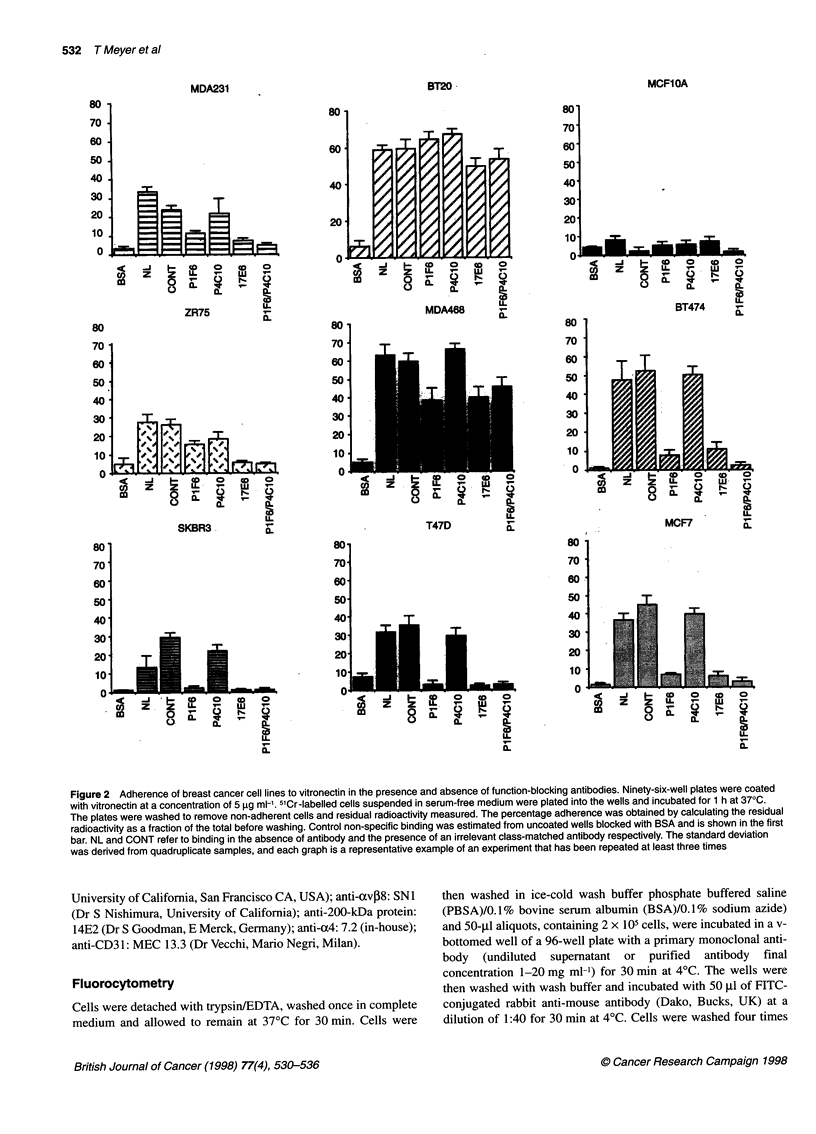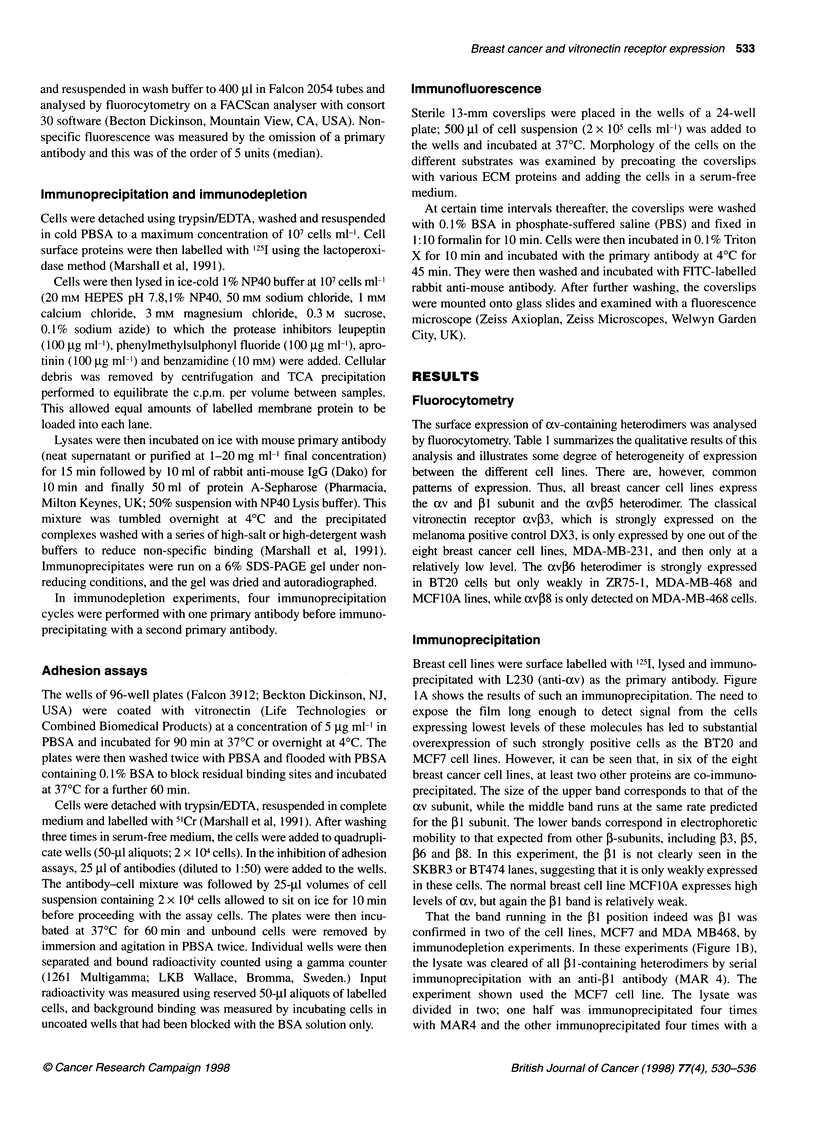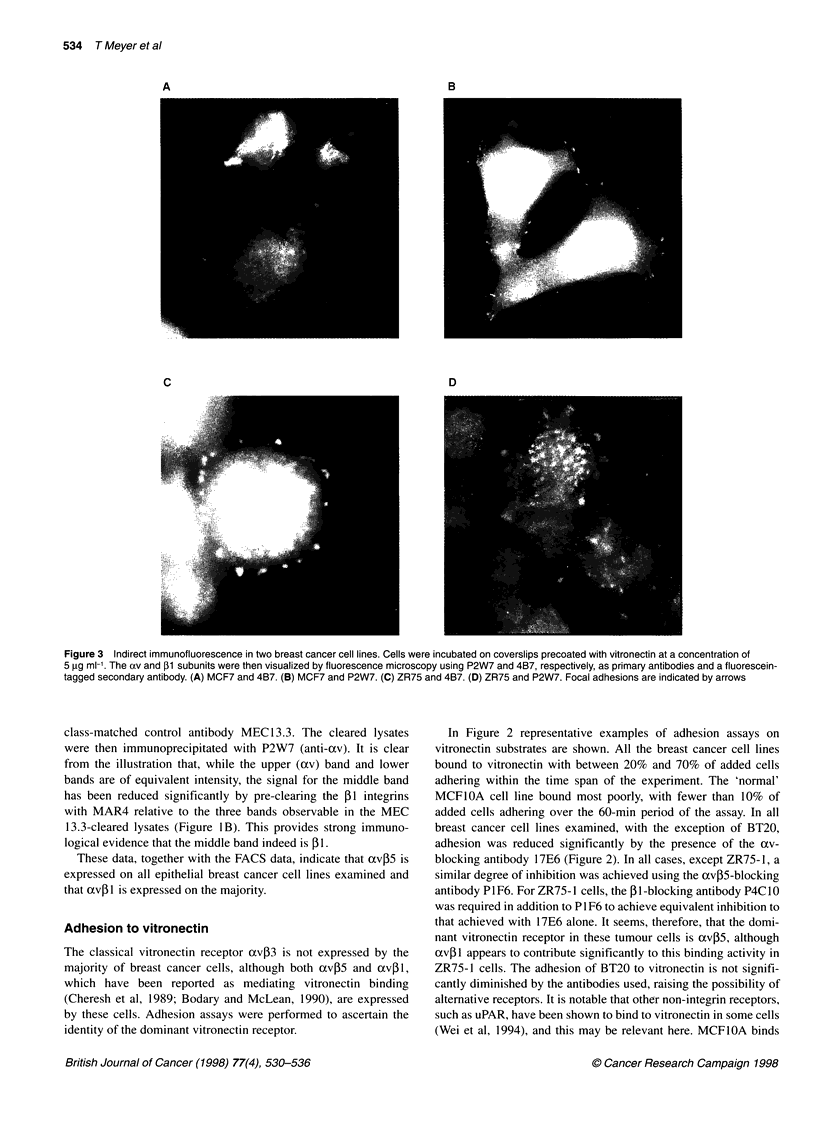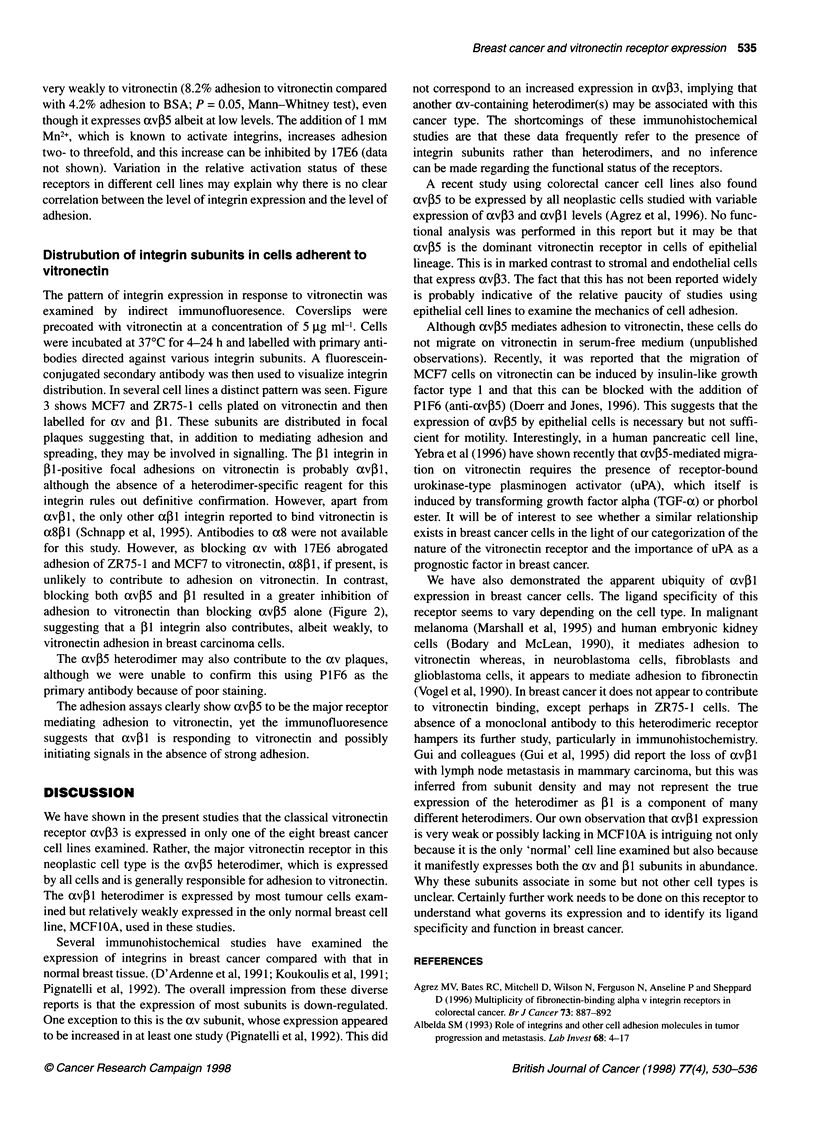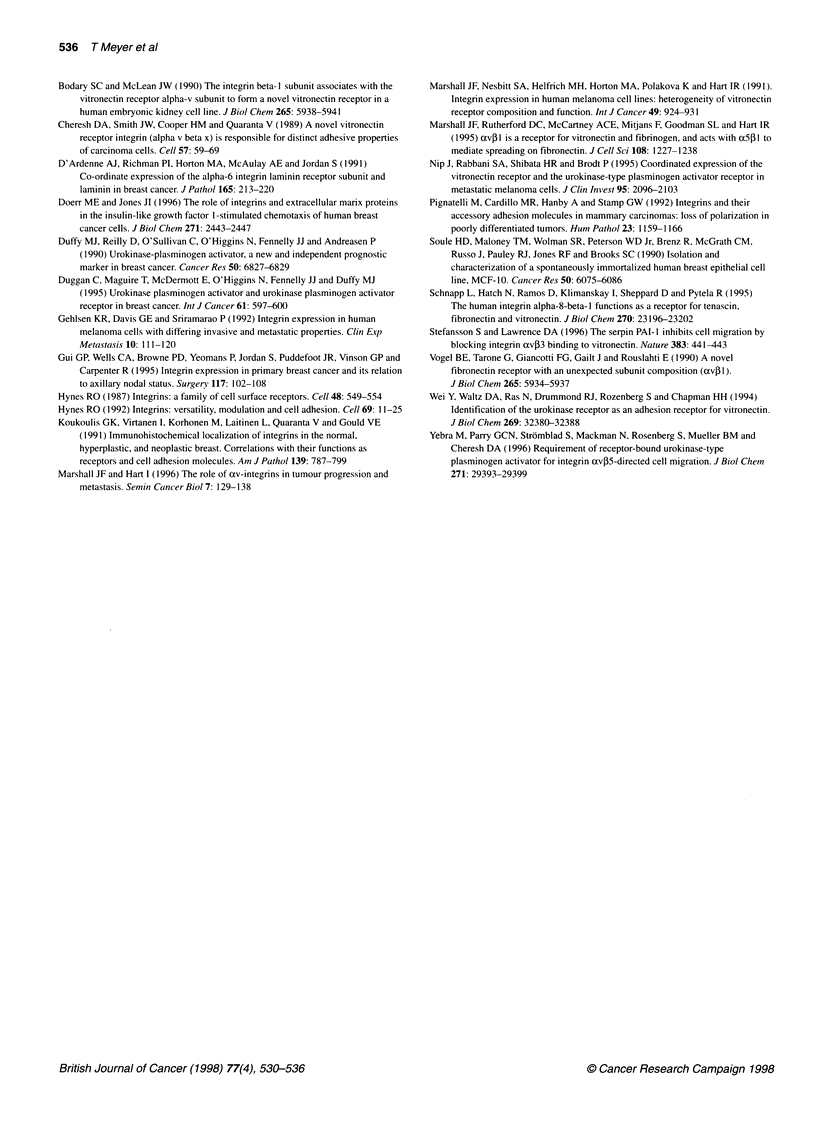# Expression of alphav integrins and vitronectin receptor identity in breast cancer cells.

**DOI:** 10.1038/bjc.1998.86

**Published:** 1998-02

**Authors:** T. Meyer, J. F. Marshall, I. R. Hart

**Affiliations:** Richard Dimbleby Department of Cancer Research, St Thomas' Hospital, London, UK.

## Abstract

**Images:**


British Journal of Cancer (1998) 77(4), 530–536
© 1998 Cancer Research Campaign

### Expression of αv integrins and vitronectin receptor identity in breast cancer cells

**T Meyer, JF Marshall and IR Hart**

Richard Dimbleby Department of Cancer Research/ICRF Laboratory, St Thomas' Hospital, Lambeth Palace Road, London SE1 7EH, UK

**Summary** In the present study we have used fluorocytometry and immunoprecipitation to characterize the expression of αv-containing integrins in a panel of eight human breast cancer cell lines and one normal human mammary epithelial line. We show that the classical vitronectin receptor αvβ3 is expressed in only one cell line (MDA-MB-231), whereas αvβ5 is expressed on all breast cancer cell lines and αvβ1 is expressed on the majority. Using adherence assays to purified ligands in the presence and absence of function-blocking monoclonal antibodies, we have demonstrated that αvβ5 mediates adhesion to vitronectin in the majority of these cells. In one cell line, ZR75-1, αvβ1 contributes significantly to adhesion to immobilized vitronectin. The formation of focal adhesions containing the αv and β1 subunits on vitronectin is also demonstrated by indirect immunofluorescence.

**Keywords:** Breast cancer; integrins; cell adhesion; vitronectin; extracellular matrix; fluorocytometry

Breast cancer affects 1 in 12 women in the UK and accounts for 18% of female malignant disease worldwide. Of those who present with apparently operable disease, more than half will die from metastatic disease. Understanding the biological mechanisms underlying the metastatic process is therefore of major importance in the hope that such understanding will lead to the evolution of new therapeutic strategies.

Metastasis requires that the disseminating cell disengages from its primary site, migrates and adheres at a distant site – all processes dependent on regulated–dysregulated cellular adhesion. Many of these functions appear to be modulated by integrins, and there is a growing body of evidence suggesting that variations in expression of these molecules can have a profound effect upon tumour biology (Albelda, 1993).

Integrins include a diverse family of heterodimeric cell surface receptors for constituents of the extracellular matrix (ECM) and occasionally for other cell-associated adhesion molecules (Hynes, 1987). They are composed of non-covalently associated α- and β-glycoprotein subunits, and receptor diversity and ligand specificity are generated by the various associations of at least eight known β-subunits and 14 α-subunits.

In recent years, it has become apparent that integrins do not function merely as transmembrane rivets, linking the cell to the ECM, but that they are also involved in signalling (Hynes, 1992). In this way, the extracellular environment can influence cellular activity during functions as diverse as migration, differentiation and cell survival.

In the present study we have examined the expression and function of the αv-containing heterodimers in a panel of breast cancer cell lines. The αv subunit has been shown to dimerize with a variety of β-subunits, including β1, β3, β5, β6 and β8 and, with the exception of αvβ6, all these heterodimers have been shown to bind to the substrate vitronectin. Some of these receptors have other ligands, most notably the promiscuous αvβ3, so that the composition of the αv-containing heterodimers can affect cell behaviour by determining adhesive interactions.

The nature of the vitronectin receptor is of particular interest in oncology because it has been implicated in tumour progression and acquisition of invasiveness (Marshall and Hart, 1996). In melanoma, for example, the expression of αvβ3 correlates with invasiveness (Gehlsen et al, 1992) and there is a correlation between αvβ3 and tumorigenic capacity (Marshall et al, 1991; Marshall and Hart, 1996). Recently, there has been evidence of a close functional inter-relationship between members of the αv subfamily and the uPA/uPAR proteolytic system (Nip et al, 1995; Stefansson and Lawrence, 1996; Yebra et al, 1996). This is of particular interest in breast cancer in which uPA (Duffy et al, 1990) and uPAR expression have been shown to correlate with prognosis (Duggan et al, 1995). Accordingly, as a first step in elucidating the role of vitronectin receptors in breast cancer, it seemed important to examine the composition and function of putative vitronectin receptors, i.e. αv-containing heterodimers, in breast cancer cells. We have used fluorocytometry, immunoprecipitation and indirect immunofluorescence to examine the expression of αv integrins in a panel of breast cancer cell lines and identified the dominant vitronectin receptor by performing adhesion assays with function-blocking antibodies.

### MATERIALS AND METHODS

### Cell lines and tissue culture

All cell lines were obtained from the Central Cell Services at the Imperial Cancer Research Fund. Eight human breast cancer cell lines were used in this study and were cultured in the following media supplemented with 10% fetal calf serum (FCS) (GIBCO BRL, Paisley, UK) and L-glutamine 4 mM. Lines ZR75-1, MDA-MB-231 and MDA-MB 468 were grown in Eagle's minimal essential medium (EMEM). Lines T47D and BT474 were grown in Roswell Park Memorial Institute medium (RPMI-1640) supplemented with

*Received 30 April 1997*
*Revised 7 July 1997*
*Accepted 14 July 1997*

*Correspondence to:* T Meyer

**Table 1** Expression of αv and β1 integrins on the surface of breast carcinoma cell lines determined by fluorocytometry

| Cell line | Integrin subunit or heterodimer (monoclonal antibody) | | | | | | | | | |
|---|---|---|---|---|---|---|---|---|---|---|
| | αv | β1 | | | αvβ3 | | αvβ5 | | αvβ6 | αvβ8 |
| | P2W7 | MAR4 | TS2/16 | 4B7 | LM609 | 23C6 | P1F6 | P3G2 | E7P6 | SN1 |
| ZR75 | ++ | $++_1$ | $++_1$ | ++ | $-_1$ | − | + | $+_1$ | + | − |
| T47D | ++ | $++_1$ | ++ | $++_1$ | − | − | + | $+_1$ | − | − |
| BT20 | ++ | $++_2$ | $++_2$ | | $-_1$ | − | ++ | | ++ | − |
| SKBR3 | ++ | | ++ | | $-_2$ | − | ++ | | − | − |
| MB468 | ++ | $++_1$ | $++_2$ | | | − | + | | + | + |
| MCF7 | ++ | $++_1$ | $++_1$ | ++ | $-_1$ | − | + | $+_1$ | − | − |
| MDA231 | ++ | | $++_1$ | $++_2$ | $+_1$ | + | + | $++_1$ | − | − |
| BT474 | + | $+_1$ | ++ | | $-_1$ | − | + | | − | − |
| MCF10A | ++ | | ++ | | $-_2$ | − | + | | + | − |
| DX3 (melanoma) | ++ | + | | | | ++ | | ++ | − | + |

Background fluorescence was measured by omitting the primary antibody, and this value was subtracted from the value obtained with the primary antibody to give a median fluorescence. Cells were considered negative (–) if the fluorescence was less than 5 units; + indicates a fluorescence of 5–50 units and ++ above 50 units. The values were derived from at least three separate experiments unless indicated by a subscript. A melanoma cell line (DX3) has been included as a positive control for αvβ3 that is absent or expressed weakly in the breast cell lines.

**Figure 1** (**A**) Immunoprecipitation of breast cancer cells. Cell-surface proteins were labelled with $^{125}$I, lysed and immunoprecipitated with a monoclonal antibody against αv (L230). L230 co-precipitates a β1 band in all the breast cell lines, in addition to a lower band corresponding in size to the other β-subunits. The composition of this band in each case can be inferred from the FACS data. (**B**) The identity of the putative β1 band is confirmed by immunodepletion in MCF7 cells that were surface labelled as above and then cleared of β1-containing heterodimers by serial immunoprecipitation with MAR4. The residual lysate was then immunoprecipitated with P2W7. Compared with the control, which was depleted with CD31, there has been a significant reduction in the intensity of the middle band confirming its identity as β1

insulin (Sigma) at 10 μg ml$^{-1}$. Line SKBR3 was grown in RPMI-1640, 10% FCS. Line MCF7 (an insulin-dependent variant) was grown in EMEM with insulin at 20 μg ml$^{-1}$ and line BT20 was maintained in MEM. MCF10A is a cell line established from the mammary tissue of a woman with fibrocystic disease and is felt to represent a normal mammary epithelial phenotype (Soule et al, 1990). This was cultured in a 1:1 mixture of EMEM and Ham's F12 medium supplemented with 5% horse serum (GIBCO), 20 ng ml$^{-1}$ epidermal growth factor (Sigma), 10 μg ml$^{-1}$ insulin, 5 mg ml$^{-1}$ hydrocortisone and 100 ng ml$^{-1}$ cholera toxin (Sigma). DX3 is a human melanoma cell line used as a positive control in some experiments and this was cultured in EMEM with 10% FCS and L-glutamine.

All cells were grown as monolayers on plastic at 37°C in a humidified atmosphere of 8% carbon dioxide–92% air. Cells were subcultured at 70–90% confluency using trypsin 0.25% (w/v)/ EDTA 5 mM to detach cells.

### Antibodies

The following mouse monoclonal antibodies were used – anti-αv: P2W7 (produced in-house), 17E6 (function blocking; a gift from Dr SL Goodman, E Merck, Germany), L230 (ATCC); anti-β1: MAR 4 (Dr S Martingnone, Istituto Nazionale per lo Studio e Curio dei Tumori, Milan, Italy), TS2/16 (ATCC) and 4B7 (in-house), P4C10 (function blocking; Life Technologies); anti-αvβ3: 23C6 (Professor MA Horton, University College London) and LM609 (Chemicon International, Harrow, UK); anti-αvβ5: P1F6 (Life Technologies), P3G2 (Dr D Cheresh, The Scripps Research Institute, La Jolla, CA, USA); anti-αvβ6: E7P6 (Dr D Sheppard,

© Cancer Research Campaign 1998

**Figure 2** Adherence of breast cancer cell lines to vitronectin in the presence and absence of function-blocking antibodies. Ninety-six-well plates were coated with vitronectin at a concentration of 5 μg ml$^{-1}$. $^{51}$Cr-labelled cells suspended in serum-free medium were plated into the wells and incubated for 1 h at 37°C. The plates were washed to remove non-adherent cells and residual radioactivity measured. The percentage adherence was obtained by calculating the residual radioactivity as a fraction of the total before washing. Control non-specific binding was estimated from uncoated wells blocked with BSA and is shown in the first bar. NL and CONT refer to binding in the absence of antibody and the presence of an irrelevant class-matched antibody respectively. The standard deviation was derived from quadruplicate samples, and each graph is a representative example of an experiment that has been repeated at least three times

University of California, San Francisco CA, USA); anti-αvβ8: SN1 (Dr S Nishimura, University of California); anti-200-kDa protein: 14E2 (Dr S Goodman, E Merck, Germany); anti-α4: 7.2 (in-house); anti-CD31: MEC 13.3 (Dr Vecchi, Mario Negri, Milan).

### Fluorocytometry

Cells were detached with trypsin/EDTA, washed once in complete medium and allowed to remain at 37°C for 30 min. Cells were then washed in ice-cold wash buffer phosphate buffered saline (PBSA)/0.1% bovine serum albumin (BSA)/0.1% sodium azide) and 50-μl aliquots, containing $2 \times 10^5$ cells, were incubated in a v-bottomed well of a 96-well plate with a primary monoclonal antibody (undiluted supernatant or purified antibody final concentration 1–20 mg ml$^{-1}$) for 30 min at 4°C. The wells were then washed with wash buffer and incubated with 50 μl of FITC-conjugated rabbit anti-mouse antibody (Dako, Bucks, UK) at a dilution of 1:40 for 30 min at 4°C. Cells were washed four times

© Cancer Research Campaign 1998

and resuspended in wash buffer to 400 µl in Falcon 2054 tubes and analysed by fluorocytometry on a FACScan analyser with consort 30 software (Becton Dickinson, Mountain View, CA, USA). Non-specific fluorescence was measured by the omission of a primary antibody and this was of the order of 5 units (median).

### Immunoprecipitation and immunodepletion

Cells were detached using trypsin/EDTA, washed and resuspended in cold PBSA to a maximum concentration of $10^7$ cells $ml^{-1}$. Cell surface proteins were then labelled with $^{125}I$ using the lactoperoxidase method (Marshall et al, 1991).

Cells were then lysed in ice-cold 1% NP40 buffer at $10^7$ cells $ml^{-1}$ (20 mM HEPES pH 7.8,1% NP40, 50 mM sodium chloride, 1 mM calcium chloride, 3 mM magnesium chloride, 0.3 M sucrose, 0.1% sodium azide) to which the protease inhibitors leupeptin (100 µg $ml^{-1}$), phenylmethylsulphonyl fluoride (100 µg $ml^{-1}$), aprotinin (100 µg $ml^{-1}$) and benzamidine (10 mM) were added. Cellular debris was removed by centrifugation and TCA precipitation performed to equilibrate the c.p.m. per volume between samples. This allowed equal amounts of labelled membrane protein to be loaded into each lane.

Lysates were then incubated on ice with mouse primary antibody (neat supernatant or purified at 1–20 mg $ml^{-1}$ final concentration) for 15 min followed by 10 ml of rabbit anti-mouse IgG (Dako) for 10 min and finally 50 ml of protein A-Sepharose (Pharmacia, Milton Keynes, UK; 50% suspension with NP40 Lysis buffer). This mixture was tumbled overnight at 4°C and the precipitated complexes washed with a series of high-salt or high-detergent wash buffers to reduce non-specific binding (Marshall et al, 1991). Immunoprecipitates were run on a 6% SDS-PAGE gel under non-reducing conditions, and the gel was dried and autoradiographed.

In immunodepletion experiments, four immunoprecipitation cycles were performed with one primary antibody before immunoprecipitating with a second primary antibody.

### Adhesion assays

The wells of 96-well plates (Falcon 3912; Beckton Dickinson, NJ, USA) were coated with vitronectin (Life Technologies or Combined Biomedical Products) at a concentration of 5 µg $ml^{-1}$ in PBSA and incubated for 90 min at 37°C or overnight at 4°C. The plates were then washed twice with PBSA and flooded with PBSA containing 0.1% BSA to block residual binding sites and incubated at 37°C for a further 60 min.

Cells were detached with trypsin/EDTA, resuspended in complete medium and labelled with $^{51}Cr$ (Marshall et al, 1991). After washing three times in serum-free medium, the cells were added to quadruplicate wells (50-µl aliquots; $2 \times 10^4$ cells). In the inhibition of adhesion assays, 25 µl of antibodies (diluted to 1:50) were added to the wells. The antibody–cell mixture was followed by 25-µl volumes of cell suspension containing $2 \times 10^4$ cells allowed to sit on ice for 10 min before proceeding with the assay cells. The plates were then incubated at 37°C for 60 min and unbound cells were removed by immersion and agitation in PBSA twice. Individual wells were then separated and bound radioactivity counted using a gamma counter (1261 Multigamma; LKB Wallace, Bromma, Sweden.) Input radioactivity was measured using reserved 50-µl aliquots of labelled cells, and background binding was measured by incubating cells in uncoated wells that had been blocked with the BSA solution only.

### Immunofluorescence

Sterile 13-mm coverslips were placed in the wells of a 24-well plate; 500 µl of cell suspension ($2 \times 10^5$ cells $ml^{-1}$) was added to the wells and incubated at 37°C. Morphology of the cells on the different substrates was examined by precoating the coverslips with various ECM proteins and adding the cells in a serum-free medium.

At certain time intervals thereafter, the coverslips were washed with 0.1% BSA in phosphate-suffered saline (PBS) and fixed in 1:10 formalin for 10 min. Cells were then incubated in 0.1% Triton X for 10 min and incubated with the primary antibody at 4°C for 45 min. They were then washed and incubated with FITC-labelled rabbit anti-mouse antibody. After further washing, the coverslips were mounted onto glass slides and examined with a fluorescence microscope (Zeiss Axioplan, Zeiss Microscopes, Welwyn Garden City, UK).

### RESULTS

### Fluorocytometry

The surface expression of αv-containing heterodimers was analysed by fluorocytometry. Table 1 summarizes the qualitative results of this analysis and illustrates some degree of heterogeneity of expression between the different cell lines. There are, however, common patterns of expression. Thus, all breast cancer cell lines express the αv and β1 subunit and the αvβ5 heterodimer. The classical vitronectin receptor αvβ3, which is strongly expressed on the melanoma positive control DX3, is only expressed by one out of the eight breast cancer cell lines, MDA-MB-231, and then only at a relatively low level. The αvβ6 heterodimer is strongly expressed in BT20 cells but only weakly in ZR75-1, MDA-MB-468 and MCF10A lines, while αvβ8 is only detected on MDA-MB-468 cells.

### Immunoprecipitation

Breast cell lines were surface labelled with $^{125}I$, lysed and immunoprecipitated with L230 (anti-αv) as the primary antibody. Figure 1A shows the results of such an immunoprecipitation. The need to expose the film long enough to detect signal from the cells expressing lowest levels of these molecules has led to substantial overexpression of such strongly positive cells as the BT20 and MCF7 cell lines. However, it can be seen that, in six of the eight breast cancer cell lines, at least two other proteins are co-immunoprecipitated. The size of the upper band corresponds to that of the αv subunit, while the middle band runs at the same rate predicted for the β1 subunit. The lower bands correspond in electrophoretic mobility to that expected from other β-subunits, including β3, β5, β6 and β8. In this experiment, the β1 is not clearly seen in the SKBR3 or BT474 lanes, suggesting that it is only weakly expressed in these cells. The normal breast cell line MCF10A expresses high levels of αv, but again the β1 band is relatively weak.

That the band running in the β1 position indeed was β1 was confirmed in two of the cell lines, MCF7 and MDA MB468, by immunodepletion experiments. In these experiments (Figure 1B), the lysate was cleared of all β1-containing heterodimers by serial immunoprecipitation with an anti-β1 antibody (MAR 4). The experiment shown used the MCF7 cell line. The lysate was divided in two; one half was immunoprecipitated four times with MAR4 and the other immunoprecipitated four times with a

© Cancer Research Campaign 1998

**Figure 3** Indirect immunofluorescence in two breast cancer cell lines. Cells were incubated on coverslips precoated with vitronectin at a concentration of 5 μg $ml^{-1}$. The αv and β1 subunits were then visualized by fluorescence microscopy using P2W7 and 4B7, respectively, as primary antibodies and a fluorescein-tagged secondary antibody. (**A**) MCF7 and 4B7. (**B**) MCF7 and P2W7. (**C**) ZR75 and 4B7. (**D**) ZR75 and P2W7. Focal adhesions are indicated by arrows class-matched control antibody MEC13.3. The cleared lysates were then immunoprecipitated with P2W7 (anti-αv). It is clear from the illustration that, while the upper (αv) band and lower bands are of equivalent intensity, the signal for the middle band has been reduced significantly by pre-clearing the β1 integrins with MAR4 relative to the three bands observable in the MEC 13.3-cleared lysates (Figure 1B). This provides strong immunological evidence that the middle band indeed is β1.

These data, together with the FACS data, indicate that αvβ5 is expressed on all epithelial breast cancer cell lines examined and that αvβ1 is expressed on the majority.

### Adhesion to vitronectin

The classical vitronectin receptor αvβ3 is not expressed by the majority of breast cancer cells, although both αvβ5 and αvβ1, which have been reported as mediating vitronectin binding (Cheresh et al, 1989; Bodary and McLean, 1990), are expressed by these cells. Adhesion assays were performed to ascertain the identity of the dominant vitronectin receptor.

In Figure 2 representative examples of adhesion assays on vitronectin substrates are shown. All the breast cancer cell lines bound to vitronectin with between 20% and 70% of added cells adhering within the time span of the experiment. The 'normal' MCF10A cell line bound most poorly, with fewer than 10% of added cells adhering over the 60-min period of the assay. In all breast cancer cell lines examined, with the exception of BT20, adhesion was reduced significantly by the presence of the αv-blocking antibody 17E6 (Figure 2). In all cases, except ZR75-1, a similar degree of inhibition was achieved using the αvβ5-blocking antibody P1F6. For ZR75-1 cells, the β1-blocking antibody P4C10 was required in addition to P1F6 to achieve equivalent inhibition to that achieved with 17E6 alone. It seems, therefore, that the dominant vitronectin receptor in these tumour cells is αvβ5, although αvβ1 appears to contribute significantly to this binding activity in ZR75-1 cells. The adhesion of BT20 to vitronectin is not significantly diminished by the antibodies used, raising the possibility of alternative receptors. It is notable that other non-integrin receptors, such as uPAR, have been shown to bind to vitronectin in some cells (Wei et al, 1994), and this may be relevant here. MCF10A binds

© Cancer Research Campaign 1998

very weakly to vitronectin (8.2% adhesion to vitronectin compared with 4.2% adhesion to BSA; $P = 0.05$, Mann–Whitney test), even though it expresses αvβ5 albeit at low levels. The addition of 1 mM $Mn^{2+}$, which is known to activate integrins, increases adhesion two- to threefold, and this increase can be inhibited by 17E6 (data not shown). Variation in the relative activation status of these receptors in different cell lines may explain why there is no clear correlation between the level of integrin expression and the level of adhesion.

### Distrubution of integrin subunits in cells adherent to vitronectin

The pattern of integrin expression in response to vitronectin was examined by indirect immunofluoresence. Coverslips were precoated with vitronectin at a concentration of 5 µg $ml^{-1}$. Cells were incubated at 37°C for 4–24 h and labelled with primary antibodies directed against various integrin subunits. A fluorescein-conjugated secondary antibody was then used to visualize integrin distribution. In several cell lines a distinct pattern was seen. Figure 3 shows MCF7 and ZR75-1 cells plated on vitronectin and then labelled for αv and β1. These subunits are distributed in focal plaques suggesting that, in addition to mediating adhesion and spreading, they may be involved in signalling. The β1 integrin in β1-positive focal adhesions on vitronectin is probably αvβ1, although the absence of a heterodimer-specific reagent for this integrin rules out definitive confirmation. However, apart from αvβ1, the only other αβ1 integrin reported to bind vitronectin is α8β1 (Schnapp et al, 1995). Antibodies to α8 were not available for this study. However, as blocking αv with 17E6 abrogated adhesion of ZR75-1 and MCF7 to vitronectin, α8β1, if present, is unlikely to contribute to adhesion on vitronectin. In contrast, blocking both αvβ5 and β1 resulted in a greater inhibition of adhesion to vitronectin than blocking αvβ5 alone (Figure 2), suggesting that a β1 integrin also contributes, albeit weakly, to vitronectin adhesion in breast carcinoma cells.

The αvβ5 heterodimer may also contribute to the αv plaques, although we were unable to confirm this using P1F6 as the primary antibody because of poor staining.

The adhesion assays clearly show αvβ5 to be the major receptor mediating adhesion to vitronectin, yet the immunofluoresence suggests that αvβ1 is responding to vitronectin and possibly initiating signals in the absence of strong adhesion.

### DISCUSSION

We have shown in the present studies that the classical vitronectin receptor αvβ3 is expressed in only one of the eight breast cancer cell lines examined. Rather, the major vitronectin receptor in this neoplastic cell type is the αvβ5 heterodimer, which is expressed by all cells and is generally responsible for adhesion to vitronectin. The αvβ1 heterodimer is expressed by most tumour cells examined but relatively weakly expressed in the only normal breast cell line, MCF10A, used in these studies.

Several immunohistochemical studies have examined the expression of integrins in breast cancer compared with that in normal breast tissue. (D'Ardenne et al, 1991; Koukoulis et al, 1991; Pignatelli et al, 1992). The overall impression from these diverse reports is that the expression of most subunits is down-regulated. One exception to this is the αv subunit, whose expression appeared to be increased in at least one study (Pignatelli et al, 1992). This did not correspond to an increased expression in αvβ3, implying that another αv-containing heterodimer(s) may be associated with this cancer type. The shortcomings of these immunohistochemical studies are that these data frequently refer to the presence of integrin subunits rather than heterodimers, and no inference can be made regarding the functional status of the receptors.

A recent study using colorectal cancer cell lines also found αvβ5 to be expressed by all neoplastic cells studied with variable expression of αvβ3 and αvβ1 levels (Agrez et al, 1996). No functional analysis was performed in this report but it may be that αvβ5 is the dominant vitronectin receptor in cells of epithelial lineage. This is in marked contrast to stromal and endothelial cells that express αvβ3. The fact that this has not been reported widely is probably indicative of the relative paucity of studies using epithelial cell lines to examine the mechanics of cell adhesion.

Although αvβ5 mediates adhesion to vitronectin, these cells do not migrate on vitronectin in serum-free medium (unpublished observations). Recently, it was reported that the migration of MCF7 cells on vitronectin can be induced by insulin-like growth factor type 1 and that this can be blocked with the addition of P1F6 (anti-αvβ5) (Doerr and Jones, 1996). This suggests that the expression of αvβ5 by epithelial cells is necessary but not sufficient for motility. Interestingly, in a human pancreatic cell line, Yebra et al (1996) have shown recently that αvβ5-mediated migration on vitronectin requires the presence of receptor-bound urokinase-type plasminogen activator (uPA), which itself is induced by transforming growth factor alpha (TGF-α) or phorbol ester. It will be of interest to see whether a similar relationship exists in breast cancer cells in the light of our categorization of the nature of the vitronectin receptor and the importance of uPA as a prognostic factor in breast cancer.

We have also demonstrated the apparent ubiquity of αvβ1 expression in breast cancer cells. The ligand specificity of this receptor seems to vary depending on the cell type. In malignant melanoma (Marshall et al, 1995) and human embryonic kidney cells (Bodary and McLean, 1990), it mediates adhesion to vitronectin whereas, in neuroblastoma cells, fibroblasts and glioblastoma cells, it appears to mediate adhesion to fibronectin (Vogel et al, 1990). In breast cancer it does not appear to contribute to vitronectin binding, except perhaps in ZR75-1 cells. The absence of a monoclonal antibody to this heterodimeric receptor hampers its further study, particularly in immunohistochemistry. Gui and colleagues (Gui et al, 1995) did report the loss of αvβ1 with lymph node metastasis in mammary carcinoma, but this was inferred from subunit density and may not represent the true expression of the heterodimer as β1 is a component of many different heterodimers. Our own observation that αvβ1 expression is very weak or possibly lacking in MCF10A is intriguing not only because it is the only 'normal' cell line examined but also because it manifestly expresses both the αv and β1 subunits in abundance. Why these subunits associate in some but not other cell types is unclear. Certainly further work needs to be done on this receptor to understand what governs its expression and to identify its ligand specificity and function in breast cancer.

### REFERENCES

Agrez MV, Bates RC, Mitchell D, Wilson N, Ferguson N, Anseline P and Sheppard D (1996) Multiplicity of fibronectin-binding alpha v integrin receptors in colorectal cancer. *Br J Cancer* **73**: 887–892

Albelda SM (1993) Role of integrins and other cell adhesion molecules in tumor progression and metastasis. *Lab Invest* **68**: 4–17

© Cancer Research Campaign 1998

Bodary SC and McLean JW (1990) The integrin beta-1 subunit associates with the vitronectin receptor alpha-v subunit to form a novel vitronectin receptor in a human embryonic kidney cell line. *J Biol Chem* **265**: 5938–5941

Cheresh DA, Smith JW, Cooper HM and Quaranta V (1989) A novel vitronectin receptor integrin (alpha v beta x) is responsible for distinct adhesive properties of carcinoma cells. *Cell* **57**: 59–69

D'Ardenne AJ, Richman PI, Horton MA, McAulay AE and Jordan S (1991) Co-ordinate expression of the alpha-6 integrin laminin receptor subunit and laminin in breast cancer. *J Pathol* **165**: 213–220

Doerr ME and Jones JI (1996) The role of integrins and extracellular marix proteins in the insulin-like growth factor 1-stimulated chemotaxis of human breast cancer cells. *J Biol Chem* **271**: 2443–2447

Duffy MJ, Reilly D, O'Sullivan C, O'Higgins N, Fennelly JJ and Andreasen P (1990) Urokinase-plasminogen activator, a new and independent prognostic marker in breast cancer. *Cancer Res* **50**: 6827–6829

Duggan C, Maguire T, McDermott E, O'Higgins N, Fennelly JJ and Duffy MJ (1995) Urokinase plasminogen activator and urokinase plasminogen activator receptor in breast cancer. *Int J Cancer* **61**: 597–600

Gehlsen KR, Davis GE and Sriramarao P (1992) Integrin expression in human melanoma cells with differing invasive and metastatic properties. *Clin Exp Metastasis* **10**: 111–120

Gui GP, Wells CA, Browne PD, Yeomans P, Jordan S, Puddefoot JR, Vinson GP and Carpenter R (1995) Integrin expression in primary breast cancer and its relation to axillary nodal status. *Surgery* **117**: 102–108

Hynes RO (1987) Integrins: a family of cell surface receptors. *Cell* **48**: 549–554

Hynes RO (1992) Integrins: versatility, modulation and cell adhesion. *Cell* **69**: 11–25

Koukoulis GK, Virtanen I, Korhonen M, Laitinen L, Quaranta V and Gould VE (1991) Immunohistochemical localization of integrins in the normal, hyperplastic, and neoplastic breast. Correlations with their functions as receptors and cell adhesion molecules. *Am J Pathol* **139**: 787–799

Marshall JF and Hart I (1996) The role of αv-integrins in tumour progression and metastasis. *Semin Cancer Biol* **7**: 129–138

Marshall JF, Nesbitt SA, Helfrich MH, Horton MA, Polakova K and Hart IR (1991). Integrin expression in human melanoma cell lines: heterogeneity of vitronectin receptor composition and function. *Int J Cancer* **49**: 924–931

Marshall JF, Rutherford DC, McCartney ACE, Mitjans F, Goodman SL and Hart IR (1995) αvβ1 is a receptor for vitronectin and fibrinogen, and acts with α5β1 to mediate spreading on fibronectin. *J Cell Sci* **108**: 1227–1238

Nip J, Rabbani SA, Shibata HR and Brodt P (1995) Coordinated expression of the vitronectin receptor and the urokinase-type plasminogen activator receptor in metastatic melanoma cells. *J Clin Invest* **95**: 2096–2103

Pignatelli M, Cardillo MR, Hanby A and Stamp GW (1992) Integrins and their accessory adhesion molecules in mammary carcinomas: loss of polarization in poorly differentiated tumors. *Hum Pathol* **23**: 1159–1166

Soule HD, Maloney TM, Wolman SR, Peterson WD Jr, Brenz R, McGrath CM, Russo J, Pauley RJ, Jones RF and Brooks SC (1990) Isolation and characterization of a spontaneously immortalized human breast epithelial cell line, MCF-10. *Cancer Res* **50**: 6075–6086

Schnapp L, Hatch N, Ramos D, Klimanskay I, Sheppard D and Pytela R (1995) The human integrin alpha-8-beta-1 functions as a receptor for tenascin, fibronectin and vitronectin. *J Biol Chem* **270**: 23196–23202

Stefansson S and Lawrence DA (1996) The serpin PAI-1 inhibits cell migration by blocking integrin αvβ3 binding to vitronectin. *Nature* **383**: 441–443

Vogel BE, Tarone G, Giancotti FG, Gailt J and Rouslahti E (1990) A novel fibronectin receptor with an unexpected subunit composition (αvβ1). *J Biol Chem* **265**: 5934–5937

Wei Y, Waltz DA, Ras N, Drummond RJ, Rozenberg S and Chapman HH (1994) Identification of the urokinase receptor as an adhesion receptor for vitronectin. *J Biol Chem* **269**: 32380–32388

Yebra M, Parry GCN, Strömblad S, Mackman N, Rosenberg S, Mueller BM and Cheresh DA (1996) Requirement of receptor-bound urokinase-type plasminogen activator for integrin αvβ5-directed cell migration. *J Biol Chem* **271**: 29393–29399

© Cancer Research Campaign 1998